# Training future clinicians in telehealth competencies: outcomes of a telehealth curriculum and teleOSCEs at an academic medical center

**DOI:** 10.3389/fmed.2023.1222181

**Published:** 2023-10-02

**Authors:** Rika Bajra, Malathi Srinivasan, Elise Cheng Torres, Tracy Rydel, Erika Schillinger

**Affiliations:** ^1^Division of Primary Care and Population Health, Department of Medicine, Stanford University School of Medicine, Stanford, CA, United States; ^2^One Health Teaching Scholars Program, Stanford CARE Internships Programs, Stanford Center for Asian Healthcare Research and Education, Department of Medicine, Stanford University School of Medicine, Stanford, CA, United States

**Keywords:** telehealth, telemedicine, competencies, medical student, curriculum development, OSCE

## Abstract

**Background:**

This study describes the program and learning outcomes of a telehealth skills curriculum based on the Association of American Medical Colleges (AAMC) telehealth competencies for clerkship-level medical students.

**Methods:**

A total of 133 third- and fourth-year medical students in a required family medicine clerkship at Stanford University School of Medicine participated in a telehealth curriculum, including a telehealth workshop, site-specific telehealth clinical encounters, and telemedicine objective structured clinical examinations (teleOSCEs) between July 2020 and August 2021. Their workshop communication and physical examination competencies were assessed in two teleOSCEs utilizing a novel telehealth assessment tool. Students' attitudes, skills, and self-efficacy were assessed through voluntary pre-clerkship, post-workshop, and post-OSCE surveys.

**Discussion:**

Most learners reported low confidence in their telehealth physical examinations [*n* = 79, mean = 1.6 (scale 0–5, 5 = very confident, SD = 1.0)], which improved post-workshop [*n* = 69, 3.3 (0.9), *p* < 0.001]; almost all (97%, 70/72) felt the workshop prepared them to see patients in the clinic. In formative OSCEs, learners demonstrated appropriate “webside manner” (communication scores 94–99%, four items) but did not confirm confidentiality (21%) or review limitations of the visit (35%). In a low back pain OSCE, most learners assessed pain location (90%) and range of motion (87%); nearly half (48%) omitted strength testing.

**Conclusion:**

Our telehealth curriculum demonstrated that telehealth competencies can be taught and assessed in medical student education. Improvement in self-efficacy scores suggests that an 80-min workshop can prepare students to see patients in the clinical setting. Assessment of OSCE data informs opportunities for growth for further development in the curriculum, including addressing visit limitations and confidentiality in telehealth visits.

## Introduction

Telemedicine, the delivery of health care remotely using telecommunication technology (1), emerged at the forefront of clinical care during the pandemic and accelerated the adoption of telemedicine education in medical schools and residencies. However, even prior to the pandemic, educators recognized the need for telemedicine education. In 2018, the Association of American Medical Colleges (AAMC) convened national telemedicine experts to define foundational skills necessary for medical students, residents, and attending physicians to provide high-quality telemedicine care (2). These cross-disciplinary and cross-continuum competencies provided a scaffold for telemedicine curricula, forming learning objectives to teach and assess telemedicine skills, such as patient-centered virtual communication, appropriate virtual physical examinations, and effective utilization of digital health tools.

While these telemedicine competencies were available for access as a pre-publication document in 2020 (and formally published in March 2021) (3). curricular implementation is currently at disparate stages across medical schools with significant curricular gaps (4, 5). One survey of 156 interns demonstrated that only 12% felt “at least moderately” prepared to conduct telemedicine visits at the start of residency (6). Although the availability of telemedicine curricula has significantly increased—American medical schools offering telemedicine education in a required or elective course increased from 58 to 90% between 2018 and 2021 (7)—efficacy studies on competency-based curricula are necessary to guide the advancement of effective teaching approaches and adequately prepare future clinicians to practice medicine in an evolving health landscape.

Telemedicine objective structured clinical examinations (teleOSCEs) provide one opportunity to assess learner outcomes in a rigorous, standardized manner. While teleOSCE development and logistics have been previously described (8, 9), outcomes data, specifically as assessment tools for the acquisition of telehealth competencies, is needed. We developed and implemented a competency-based telemedicine curriculum for medical students in the family medicine core clerkship at a large academic medical center. In this study, we describe the program and learning outcomes of a telemedicine curriculum, including a skills workshop, patient care, and two teleOSCEs with over 100 medical students. The teleOSCE assessments were built around AAMC's cross-disciplinary telemedicine competencies; discipline-specific competencies were drawn from the Society of Teachers of Family Medicine (STFM) telemedicine learning objectives (10). The OSCEs utilized a novel assessment tool for telehealth encounters modified from the Kalamazoo Essential Elements Communications Checklist-Adapted (KEEC-A) (11).

## Pedagogical framework(s), competencies, and standards underlying the educational activity

We developed the following two curricular interventions: (1) an interactive telehealth workshop (Beyond Bricks and Mortar) on the first day of the required family medicine clerkship and (2) a two-station formative video-based teleOSCE administered during the last week of the clerkship. We developed the curriculum attending to core education principles, including time neutrality, multimodal learning strategies, and feedback or reinforcement with simulated and actual patients.

### Beyond bricks and mortar: a skills workshop for virtual visits

**Figure 1:**
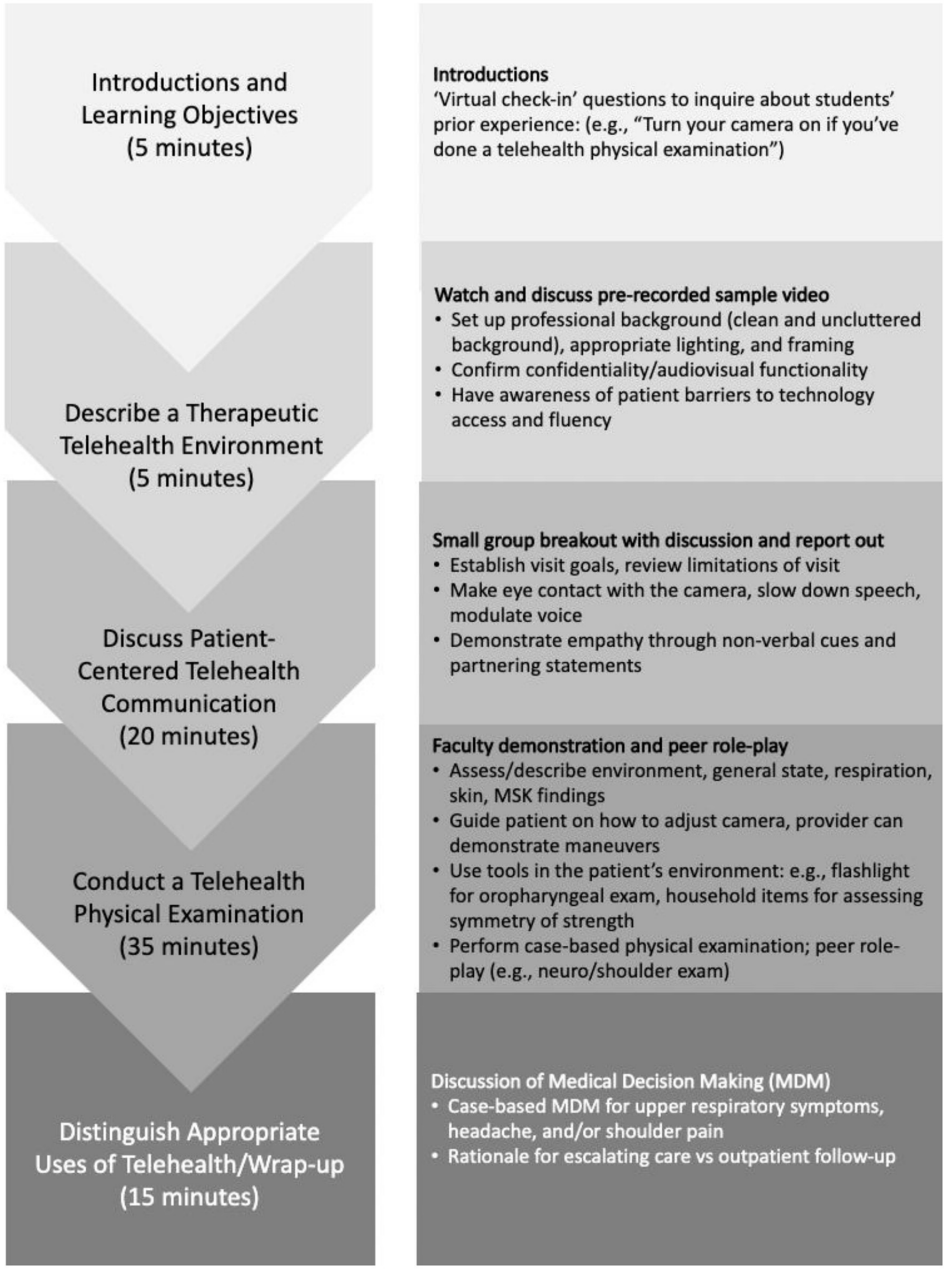
Structure of an 80-min competency-based telehealth workshop at an academic medical center. MSK: musculoskeletal.

The Beyond Bricks and Mortar telehealth workshops, launched in June 2020, were jointly taught by two faculty members with 4–6 years of telehealth clinical experience. Anchored in AAMC (3) and STFM (10) telemedicine learning objectives (Table 1), the workshops optimized interactivity through group discussions and hands-on practice. The session participants ranged from 6 to 10 students based on clerkship enrollment. The 80-min workshop (see Figure 1 for format) was taught via Zoom video conferencing software (Zoom Video Communications, San Jose, California). Telehealth communication competencies were taught through faculty-led group discussions. The workshop's learning objectives (e.g., describe a therapeutic telemedicine environment, demonstrate patient-centered telemedicine communication, conduct a telemedicine physical examination, and distinguish appropriate clinical uses of telemedicine) and format (time spent on each topic) are depicted in Figure 1.

**Table 1:** Competency domains and observable behaviors for telehealth objective structured clinical evaluations (OSCEs).

**Competency domain**	ACGME^**^core competency and sub-competencies	AAMC telehealth competency/STFM^***^learning objective for medical school graduate	**Teaching method in didactic**	**Observable behaviors (OSCE checklist)**
Communication via telehealth (AAMC^*^)	Interpersonal and communication skills: create and sustain a therapeutic relationship with patients and families	Develops an effective rapport with patients via (real or simulated) video visits, attending to eye contact, tone, body language, and non-verbal cues	Think/pair/share activity: methods to demonstrate empathy via verbal and non-verbal cues. Discussion of sample language and non-verbal communication (e.g., eye contact, leaning in, slowing down the pace of speech)	• Eye contact: enough to build connection, verbalizes activities (taking notes) • Uses tone/pace and posture, showing care and concern • Pays attention to verbal and non-verbal cues • Elicits and addresses emotional content
	Professionalism: demonstrates professional conduct and accountability	Assesses the environment during (actual or simulated) video visits, attending to attire, disruptions, privacy, lighting, sound, etc.	Video clip of student interviewing a student-standardized patient: reflective discussion on what was done well and areas of opportunity to improve	• Assists patients with technology as needed • Confirms confidentiality: location/participant • Appears professional in attire/background
Data collection and assessment via telehealth (AAMC)	Patient care and procedural skills: gathers essential and accurate information, counsels patients and family members, provides effective health management, maintenance, and prevention guidance	Conducts appropriate physical examination or collects relevant data on clinical status during a (real or simulated) telehealth encounter, including guiding the patient or tele-presenter	Faculty demonstrates best practices in virtual physical examination (techniques, clarity of communication, and using tools in patients' environment). Students participate in role-play in conducting a clinically directed patient self-examination of their peers	Physical examination: • Determines location of back pain • Evaluates range of motion for low back exam • Provides clarity of instruction in guiding patient through the physical examination
Medical decision-making (STFM)	Patient care and procedural skills: makes informed diagnostic and therapeutic decisions	Explains how medical decision-making may be affected by the provision of care at a distance using telehealth (e.g., how limited vital signs, physical examination, and point-of-care testing may impact decision-making)	Case-based learning: students conduct an appropriate physical examination based on clinical history and determine appropriate medical decision-making	• Reviews limitation of visit • Checks for mutual understanding of diagnostic and/or treatment plans • Reviews red flags for urgent symptoms • Clarifies follow-up arrangement

* AAMC: Association of American Medical Colleges. http://www.aamc.org/data-reports/report/telehealth-competencies.

** ACGME: Accreditation Council for Graduate Medicine Education: https://knowledgeplus.nejm.org/blog/exploring-acgme-core-competencies/.

*** STFM: Society for Teachers of Family Medicine. https://www.stfm.org/telemedicinecurriculum.

After watching a video clip of a student-standardized patient (SP) telehealth encounter, learners responded to the following reflection prompts: (1) strategies to create a therapeutic environment during virtual encounters, (2) how to demonstrate empathy with distressed patients, and (3) assess contexts where telehealth may provide value-added options compared to office visits (e.g., patients with mobility challenges). The strategies and techniques for effective communication techniques were discussed; this included assessing patients' barriers to technology utilization and setting up a professional and therapeutic environment. The faculty taught learners to incorporate best practices in “webside manner” (e.g., optimizing eye contact, modulating tone of voice), ensuring confidentiality (e.g., patient privacy and safety, use of virtual backgrounds, presence of other family members), and leveraging verbal and non-verbal cues to empathetically address an emotional patient via video.

The AAMC's telehealth competencies for data collection and assessment1 (Table 1) were taught by reviewing potential health risks in patients' environment, performing visual medication reconciliation, and reviewing best practices in virtual physical examinations, including guiding patients through examination maneuvers, and utilizing common household items (such as flashlight for oropharyngeal exam, milk gallon to assess strength) to facilitate examination (8). Learners practiced with each other via role-play with physical examination scripts and received real-time coaching from faculty. The role-play via videoconference simulated virtual physical examinations. Students conducted a virtual physical exam, considered its feasibility (12, 13), and directly experienced how clarity of instructions related to a higher quality of physical examinations. Students synthesized history and physical examination findings into their medical decision-making, including discussing rationales for escalating care, recommending in-person visits, and arranging interval follow-ups (Figure 1 details the workshop sequence.).

### Telehealth OSCE

Two teleOSCEs were administered remotely at the clerkship's conclusion via Zoom. Standardized Patients (SPs) were trained for 6 h on portraying two virtual cases and providing structured feedback. The teleOSCEs covered AAMC telehealth competencies, including patient safety, appropriate utilization, communication, data collection and assessment, and technology. Proctors included two core faculty members (clerkship directors) and two telehealth-trained rotating family medicine residents from O'Connor-Stanford Family Medicine Residency. After teleOSCE orientation, students and SPs were placed in breakout rooms for the 20-min case simulation. Students received a clinical prompt or case description via “share screen”.

The two teleOSCEs included the following: (1) chronic care management for diabetes mellitus (DM) and (2) acute, undifferentiated low back pain (LBP). For the DM teleOSCE, the SP's microphone was initially muted, requiring the student to recognize the technology barrier and provide guidance to unmute. Students navigated defusing the patient's frustration with technology while establishing rapport. To evaluate the patient with localized, non-radiating LBP, students were expected to differentiate lumbar strain from more serious diagnoses by assessing red flag symptoms and key physical examination findings, including pain location, range of motion, and strength testing (Table 2).

**Table 2:** Selected examples from medical student performance on two telehealth OSCE stations for chronic and acute conditions: proficiency and representative faculty comments.

**Objective structured clinical evaluation**	**Faculty ratings**		**Representative faculty feedback (open-ended responses)**	
**Clinical focus**	**Done**	**Needs improvement**	**Done**	**Needs improvement**
**Case 1: diabetes mellitus**				
Appears professional: attire/background	113 (93%)	8 (7%)	“We appreciate that you wore your white coat, which created a very professional first impression”	“Unmade bed was visible in the background: this can decrease the professionalism in your encounter”
Assists patient with technology as needed: camera/audio/lighting	102 (83%)	21 (17%)	“Navigated patient through unmute button”	“Could try to help her find the mute button by giving her instructions”
Confirms confidentiality: location/participants	26 (21%)	97 (79%)	“Done”	“Over telehealth, it is important to ask who else may be in the room or part of the visit”
Establishes mutual goals/agenda for the visit	104 (86%)	17 (14%)	“Skillfully navigated multiple concerns and established patient's priorities”	“One way to do this is: ‘Is there anything else you want to make sure we reviewed?”'
Reviews limitations of visit and obtains consent	42 (35%)	78 (65%)	“Excellent-most students don't do this!”	“Recommend reviewing potential limitations and obtaining consent to move forward”
Establish initial rapport	122 (99.2%)	1 (0.8%)	“Diffused patient's frustration with tech and built rapport readily”	“Consider spending a little more time in the beginning investing in the relationship by connecting to the patient in some way.”
Eye contact: enough to build a connection with the patient and verbalize off-screen activities (taking notes, reviewing chart)	117 (95%)	6 (5%)	“<Student name> explained that she will be taking notes during the encounter, explaining off-screen activities”	“Eye contact was angled downward and to your left; we would recommend moving the patient's video closer to your computer's camera”
Uses tone/pace and posture, showing care and concern	119 (98%)	3 (2%)	“Well-paced and caring tone of voice”	“We'd recommend slowing down the pace of speech or pausing periodically”
Pays attention to verbal and non-verbal cues	119 (97%)	4 (3%)	“<Student name> was attuned to the patient's facial expression and clarified when the patient seemed confused”	“Could have responded a bit more fully to the ‘chief concern' about his father's health”
Elicits and addresses emotional content	108 (90%)	12 (10%)	“Excellent response to patient explaining multiple stressors”	“We would recommend exploring the patient's life situation and how these challenges pose an obstacle for behavior modification”
**Case 2: low back pain**				
Evaluated range of motion	39 (86.67%)	6 (13.33%)	“<Student> had patient turn sideways to assess flexion/extension: this provided a much better angle”	“Better to do this with patient standing rather than sitting, especially when you will get him up to walk anyway”
Determined location of pain (paraspinal vs. spinal)	40 (88.89%)	5 (11.11%)	“Loved the explanations- ‘knobs' of the vertebrae, ‘guitar strings' of the paraspinals”	“Could try to get the patient to point or find specifically where the maximal point of tenderness is”
Assessed strength testing (toe/heel walk)	24 (53.33%)	21 (46.67%)	“<Student> recommended that the patient angle his camera downwards to see better”	“Had patient get out of the chair but did not do toe/heel walk”
Provided clarity of instruction in guiding the patient through the physical exam	42 (93.33%)	3 (6.67%)	“<Student> demonstrated the maneuvers herself and partnered with the patient”	“Might want to advise the patient to tilt the camera to see the maneuvers fully”

Comments from four faculty members on summary PDF sent to students at an academic medical center. Comments have been lightly edited for anonymity and grammar.

Faculty observers turned off their cameras and microphones and assessed students based on a checklist, while students selected “hide non-video participants” settings to simulate a one-on-one telehealth encounter. Immediately after their encounter, students received feedback from both SPs and later received faculty teleOSCE scores and feedback. Post teleOSCE, the faculty led an interactive debrief on communication skills, physical examination, medical management, and patient counseling.

### Assessment

We administered voluntary pre- and post-surveys (Qualtrics, Provo, UT) before and immediately after the telemedicine workshop, as well as immediately following the OSCE test. We assessed the learners' prior virtual patient care experiences and telehealth training, self-efficacy in virtual physical exams, and their communication skills on a 6-point scale (0 = not at all confident, 5 = extremely confident). Their perception of quality of care in a telehealth encounter compared to an in-person visit was assessed as “In-person encounters always provide better quality of care than video visits” (where 1, 2 = strongly disagree/disagree; 3 = undecided; 4,5 = agree/strongly agree).

Trained course faculty members assessed each learner's teleOSCE performance in communication and physical examination with opportunities for open-ended comments, including areas of strengths and improvement. We modified the Kalamazoo Essential Elements Communication Checklist Adapted (KEEC-A), a validated measure of physician communication skills (14), which we tailed for use in virtual encounters (see Appendix 1 for full checklist). The 30-item checklist assessed patient-centered use of technology (nine items), verbal and non-verbal cues to facilitate effective virtual communication (seven items), information-gathering (five items), and shared decision-making, including discussion of limitations of virtual visits (nine items), on a bimodal rating of “Done” or “Needs Improvement/Not Done”. The LBP checklist included four physical examination items (range of motion, localizing pain, strength, and instruction clarity), which were moved from open comments to the main checklist in December 2020. Faculty completed their formative assessments on Qualtrics, providing written feedback on areas of strength and improvement, and students received a PDF copy of the feedback. We examined univariate and bivariate outcomes using Excel v16.53 and SPSSv27.0.1.

## Learning environment (setting, students, faculty), learning objectives, and pedagogical format

### Setting

All 133 third- and fourth-year medical students, as part of their required family medicine core clerkship at the Stanford School of Medicine, participated in the telehealth didactic curriculum and 2 teleOSCEs from July 2020 to August 2021. The students were placed at 6–10 family medicine sites where they participated in various telehealth patient encounters, depending on site constraints and patients served (e.g., rural, homeless). A voluntary clerkship orientation survey demonstrated that 70% (64/92) of students reported providing patient care via telehealth encounters, but only one-third (28/92) had received any prior telehealth training. Based on the dual need to provide telehealth training and assess telehealth competencies, we developed the following two curricular interventions as described above: (1) an interactive telehealth workshop (Beyond Bricks and Mortar) on the first clerkship day and (2) a two-station formative video-based teleOSCE in the last clerkship week. We trained additional teleOSCE proctors in feedback techniques. Additionally, students shared their viewpoints about their telehealth skills and attitudes through voluntary pre-clerkship, post-workshop, and post-OSCE surveys.

Though traditionally offered as a 4-week clerkship, the family medicine clerkship transitioned to 3 weeks from July 2020 to June 2021 to accommodate students impacted by canceled rotations due to the pandemic. The Stanford University Institutional Review Board determined this was not human subjects research (Protocol 59034).

## Results

### Student's telehealth self-efficacy and viewpoints

Most clerkship students (84/121, 69%) had cared for patients via telehealth during their clerkship, with half (42/84, 50%) encountering more than half of their patients through telehealth. The students reported low baseline telehealth physical examination confidence [*n* = 78, mean = 1.6 (standard deviation 1.0)], which improved post-workshop [69, 3.3 (1.0), *p* < 0.001]. Initial confidence in telehealth communication skills was higher [85, 3.5 (1.03)] and trended toward improvement post-workshop [68, 4.0 (0.65), *p* < 0.01]. Paired data with a smaller subset of students reflected similar results. Nearly all students (70/72, 97%) felt that the workshop prepared them to see patients in a telehealth setting. Post-workshop, students shared “take-home points” in open-response comments (Table 3).

**Table 3:** Qualitative feedback from students post-telehealth workshop and post-telehealth OSCE.

	**Representative medical student responses**
**Post “Bricks and Mortar” workshop responses**	
Lessons learned	“Elements of the physical exam can be performed in a telehealth visit that provide confidence for medical decision making. The virtual visit can be a reliable primary care tool for dealing with many patient complaints and issues. The virtual sessions require a new way of thinking about clinical encounters to ensure professional, private, and effective communication with patients.” “State your confidential setting and ask patients if they feel comfortable. Be as explicit as possible with wrap-up, summary, and follow-up for the patient as they won't be checking out.” “Ask patients to bring their medications, masks, or other therapeutics and demonstrate their use.” “Finding creative ways to help patients rate their physical exam findings using standards (e.g., does a lump feel hard like bone, firm like the heel of one's palm, or soft like the abdomen?) Document your findings thoroughly since your note might be taken with some skepticism by providers who see that the exam was done virtually; make sure it's clear why you made your conclusions and what your patient actually found.”
**Post-OSCE responses**	
Benefits of telehealth	“Way more efficient. Allows more points of contact with the patient to touch base and check-in. For management of chronic conditions, I think this is much more important. When patients come all the way to the office for an in-person visit, I think they feel much more pressured to get everything medical-related in a short period. They can feel rushed, haven't had all their answers questioned, etc.” “I think video visits have a very important role in healthcare. They are much more accommodating to patients, especially when doing follow-ups or when a physical exam is not needed in managing care. It is also accommodating for individuals with disabilities, who may find that clinics are not as accommodating or have issues with transportation. I think they are also great for motivational interviewing and checking in with patients more frequently.” “Able to see patients at home, which may reveal barriers to health, and allow for discussion with family members.”
Areas of interest for further telehealth training	“I would be curious to learn more about devices being developed to help patients/providers with certain areas of telemedicine screening (i.e., at-home blood pressure cuff devices or add-ons to help with eye screening).” “Working on triage, I think, is really important; where should the patient go—should they come to the clinic, go to urgent care, go to ED? When can we order tests and imaging-based just on history without physical examination?” “Maybe some advice on what to chart and how we can chart a visit effectively—would be helpful to understand sort of what has to happen on the video visit re: can't miss things.” “I would have liked more training on discussing sensitive topics virtually. Some mental health check-ins are done virtually, and I am not comfortable discussing potential suicidal ideation on video.” “I would like to learn more about exploring musculoskeletal complaints via telehealth and learn about when are the optimal uses of scheduling a telehealth visit vs. an in-person visit.”

Prior to our workshop, students perceived telehealth encounters as inferior to in-person visits. Initially, just 17% (14/83) of students disagreed with the statement “In-person encounters always provide better quality of care than video visits”, which increased post-OSCE to 31% (37/121, *p* = 0.02) toward the clerkship conclusion, indicating that telehealth training and care positively influenced students' perceptions of quality of care in telehealth encounters. Paired learner data showed more positive effects, with 13% (8/61) of students disagreeing at baseline while 41% (25/61) of students disagreed post-OSCE, unaffected by rates of telehealth clinical encounters.

### OSCE results

The faculty used checklists with binary options (Done or Needs Improvement), in addition to open text comment boxes, to assess students. Clerkship medical students demonstrated consistently appropriate “webside manner”, including professional appearance and background (113/121, 93%), establishing initial rapport (122/123, 99%), eye contact (117/123, 95%), and tone and vocal pace (119/122, 98%). However, they did not exhibit more nuanced aspects of communication, including confirming telehealth patient's privacy and confidentiality (26/123, 21%), reviewing telehealth limitations and obtaining consent (42/120, 35%), and summarizing and reviewing red flags for follow-up (63/117, 54%). For the LBP teleOSCE, students consistently determined the location of pain (71/79, 90%), assessed range of motion (69/79, 87%), and provided clear instructions for examination maneuvers (74/79, 94%), but nearly half of the students omitted strength testing (38/79, 48%). Faculty noted areas for learner improvement, including communication and physical examination suggestions (Table 2).

Students reflected on potential telehealth benefits and challenges (Table 3), describing physical examination, building rapport, and technical issues as the most commonly encountered challenges and indicating interest in further training in physical examination techniques.

## Discussion

Our telehealth curriculum demonstrated that telehealth competencies can be taught and assessed in medical student education; improvement in self-efficacy scores suggests that an 80-min workshop can prepare students to see patients in the clinical setting. The teleOSCEs enabled faculty to provide direct feedback on telehealth competencies, while students received hands-on experience in autonomously managing telehealth encounters in a formative setting. Most students demonstrated proficiency in “webside manner” but did not address visit limitations, potentially reflecting uncertainty regarding telehealth safety and appropriateness, which suggests a need for further training in this area. Learners' viewpoints evolved after the structured curriculum coupled with simulated telehealth, as reflected by a positive shift in learner attitudes toward quality of care in telehealth encounters.

**Figure 2:**
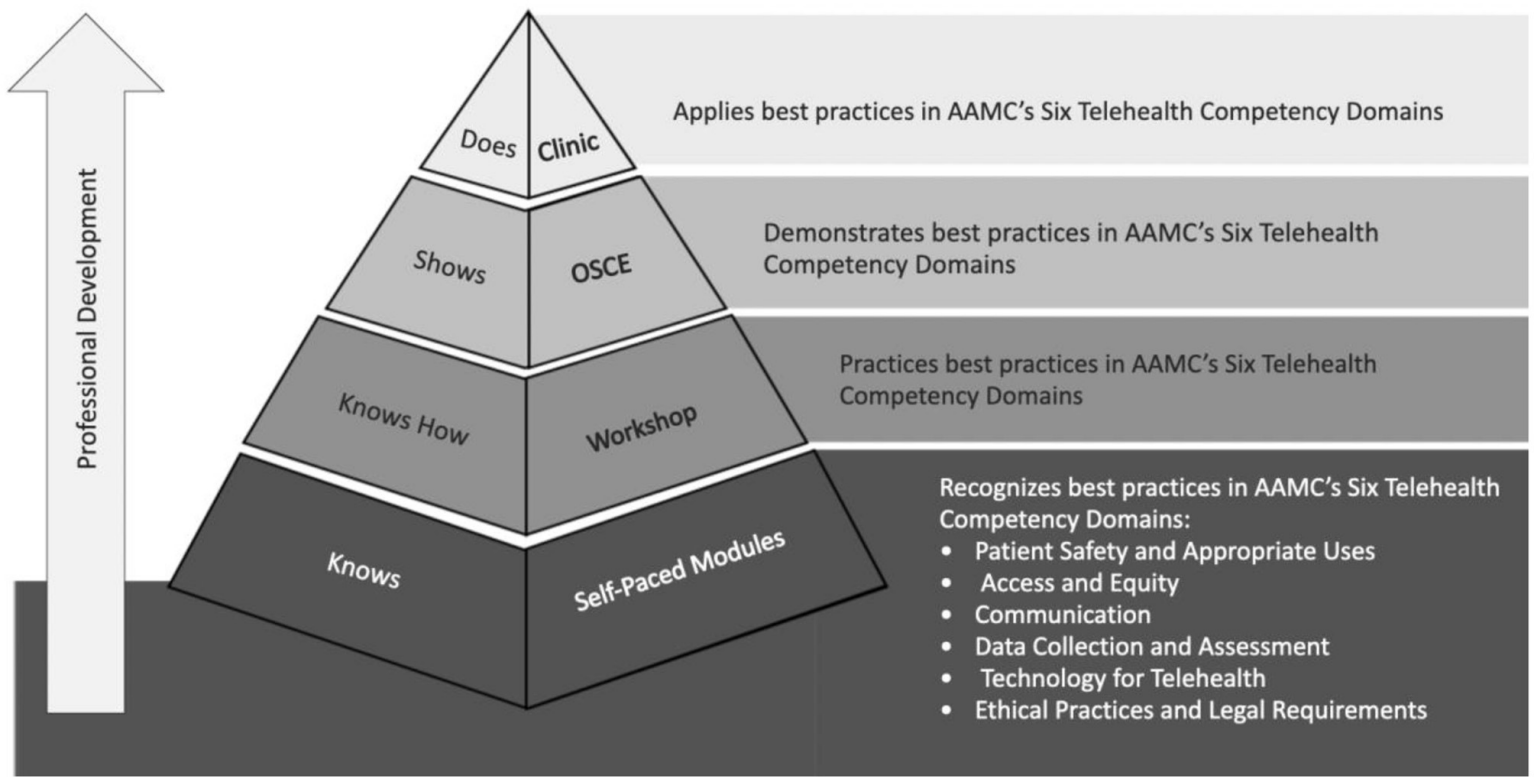
Optimizing teaching the AAMC's six telehealth competency domains through the lens of Miller's pyramid.

The emergence of telehealth offers unparalleled opportunities to enhance the quality of patient care and create more meaningful relationships with patients in their home environment. The success of telehealth depends on the successful adoption and integration of telehealth competencies into existing medical school curricula. Figure 2 demonstrates a strategy for teaching AAMC's six telehealth competency domains (3) through the lens of Miller's pyramid (15). Building on foundational competencies through asynchronous modules (10, 16), synchronous “live” workshops, like those described in our study, prioritize advanced communication skills, in-depth physical examination training, and case-based decision-making. Given our current reality in which medical students are often providing virtual patient care with little or no training, there is an urgency in developing effective telehealth curricula to teach and assess novel skills requisite to practicing medicine in health systems that will increasingly integrate telemedicine and digital health tools into clinical care.

## Limitations

Our study had several limitations. The curriculum was designed and implemented at a single institution. However, the technologies were broadly utilized, and the curriculum was based on telehealth competencies that are generalizable. Some students did not have opportunities to reinforce teachings through clinical practice, potentially minimizing the effect on self-efficacy.

## Data availability statement

The original contributions presented in the study are included in the article/Supplementary material, further inquiries can be directed to the corresponding author.

## Author contributions

MS performed the statistical analysis. RB wrote the first draft of the manuscript. All authors contributed to the conception and design of the study, manuscript revision, and read and approved the submitted version.
