# Supplementary material for: Training future clinicians in telehealth competencies: outcomes of a telehealth curriculum and teleOSCEs at an academic medical center
Source: Front Med (Lausanne). 2023 Oct 2;10:1222181. doi: 10.3389/fmed.2023.1222181 (PMC10577422; doi:10.3389/fmed.2023.1222181)
Supplement: Supplementary file 1 [file Data_Sheet_1.docx]

**Appendix: OSCE Assessment Checklist: Modified Kalamazoo for Telehealth Encounters***

| **Opening Visit  (9 items)** |
| --- |
| - Introduces self, role as medical student |
| - Greets and shows interest in patient as person, uses patient’s name |
| - Assists patient with technology as needed: camera/audio/lighting |
| - Confirms confidentiality: location/participants |
| - Allows patient to complete opening statement w/o interruption |
| - Asks to elicit full set of concerns |
| - Establishes mutual goals/agenda for visit |
| - Reviews limitations of visit, obtains consent |
| - Appears professional: attire/background |
| **Building the Relationship (7 items)** |
| - Establishes initial rapport |
| - Eye contact: looking at camera, enough to build connection, verbalizes activities (chart review, taking notes) |
| - Uses tone/pace, and posture showing care and concern |
| - Pays attention to verbal and non-verbal cues |
| - Elicits and addresses emotional content |
| - Avoids technical jargon |
| - Demonstrates confidence/appears competent |
| **Information Gathering (5 items)** |
| - Begins with open-ended questions, i.e., “Tell me more about…” |
| - Collects information in an organized manner |
| - Clarifies details as necessary w/more specific “yes/no” questions |
| - Summarizes and checks accuracy with patient |
| - Transitions effectively to additional questions |
| **Shared Decision-Making/Closing the Encounter (9 items)** |
| - Elicits patient’s chief concern, explored patient’s belief/expectations about illness |
| - Asks about events/circumstances, other people that may affect health |
| - Responds explicitly to patient statements about ideas, feelings, values |
| - Includes patient in choices and decisions to the extent s/he desires |
| - Checks for mutual understanding of diagnostic and/or treatment plans (i.e., uses teach-back method) |
| - Asks if patient has questions or concerns |
| - Summarizes/reviews red flags for urgent symptoms |
| - Clarifies follow-up or contact arrangements, i.e., where to locate patient education materials/after visit notes |
| - Acknowledges patient and provides closure to interview |
| **Physical Examination for Low Back Pain case (4 items)** |
| - Determined location of pain: spinal vs paraspinal |
| - Evaluated range of motion (flexion/extension/lateral bending/rotation) |
| - Assessed strength testing: toe/heel walk, rising out of chair |
| - Provided clarity of instruction in guiding patient through the physical examination |
| **Open ended comments** |
| ** Faculty ratings for each item are “done” or “not done/needs improvement” with an open comments column.  Students receive a PDF copy of the faculty assessment and comments.* |
